# Identification of microRNA biomarkers simultaneously expressed in circulating extracellular vesicles and atherosclerotic plaques

**DOI:** 10.3389/fcvm.2024.1307832

**Published:** 2024-04-25

**Authors:** Florian Brandes, Agnes S. Meidert, Benedikt Kirchner, Mia Yu, Sonja Gebhardt, Ortrud K. Steinlein, Michael E. Dolch, Barbara Rantner, Nikolaos Tsilimparis, Gustav Schelling, Michael W. Pfaffl, Marlene Reithmair

**Affiliations:** ^1^ Department of Anesthesiology, LMU University Hospital, LMU Munich, Munich, Germany; ^2^ Division of Animal Physiology and Immunology, School of Life Sciences Weihenstephan, Technical University of Munich, Freising, Germany; ^3^ Department of Anaesthesiology, InnKlinikum Altötting, Altötting, Germany; ^4^ Institute of Human Genetics, LMU University Hospital, LMU Munich, Munich, Germany; ^5^ Department of Vascular Surgery, LMU University Hospital, LMU Munich, Munich, Germany

**Keywords:** atherosclerosis, plaque tissue, carotid artery stenosis, microRNA expression profile, circulating extracellular vesicles

## Abstract

**Background:**

Atherosclerosis is a widespread disorder of the cardiovascular system. The early detection of plaques by circulating biomarkers is highly clinically relevant to prevent the occurrence of major complications such as stroke or heart attacks. It is known that extracellular vesicles (EVs) are important in intercellular communication in atherosclerotic disorders and carry many components of their cells of origin, including microRNAs (miRNAs). In this study, we test the assumption that miRNAs present in material acquired from plaques in patients undergoing surgery for atherosclerotic carotid artery stenosis are also expressed in circulating EVs obtained from the identical patients. This would allow the adoption of a liquid biopsy approach for the detection of plaques.

**Methods:**

We studied 22 surgical patients with atherosclerotic carotid arterial stenosis and 28 healthy controls. EVs were isolated from serum by precipitation. miRNA expression profiles of serum-derived EVs were obtained by small RNA sequencing and in plaque material simultaneously acquired from patients. A comparative analysis was performed to identify circulating atherosclerosis-associated miRNAs that are also detectable in plaques.

**Results:**

Seven miRNAs were found to be differentially regulated in patient serum compared with the serum of healthy controls. Of these, miR-193b-5p, miR-193a-5p, and miR-125a-3p were significantly upregulated in patients compared with that in healthy controls and present in both, circulating EVs and plaque material. An overrepresentation analysis of experimentally validated mRNA targets revealed an increased regulation of inflammation and vascular growth factors, key players in atherosclerosis and plaque formation.

**Conclusion:**

Our findings suggest that circulating EVs reflect plaque development in patients with symptomatic carotid artery stenosis, which can serve as biomarker candidates for detecting the presence of atherosclerotic plaques.

## Introduction

Atherosclerosis is a systemic inflammatory disorder of the arteries characterized by atherosclerotic plaque formation due to the accumulation of lipids, inflammatory cells, apoptotic cells, calcium, and extracellular matrix proteins in and at the vascular wall. When plaques become mechanically unstable, they are predisposed to plaque rupture, resulting in thrombosis and acute occlusive syndromes.

The early detection of plaques by circulating biomarkers is highly clinically relevant, but currently available marker compounds are mostly based on the detection of local or systemic inflammation and lack sensitivity and specificity (1). Although atherosclerosis is a widespread disorder of the cardiovascular system, current biomarker candidates are only modestly sensitive to indicate an increased risk for plaque presence. Furthermore, especially unstable plaques often cause only minor stenosis with few clinical symptoms, and angiography has only limited sensitivity in detecting even a mild degree of arterial stenosis (2).

Extracellular vesicles (EVs) are released by almost all cell types into their peri- and extracellular space and finally into the vascular system (3). EVs contain many components of their origin, including RNA, as well as DNA, lipids, and cytosolic and cell-surface proteins (4) and play an important role in atherosclerotic disorders (5–8) that includes the orchestration of the immune response during systemic inflammation associated with atherosclerosis (9, 10). Mechanistic studies indicate that distinct biological signals may be transported by EVs from their origin to specific target cells, indicating an important function of EVs for intercellular communication (11). It is therefore likely that EVs may also serve as transport vehicles for microRNAs (miRNAs) present in atherosclerotic plaques (12).

In this study, we assume that miRNAs that are detectable in serum-derived EVs are also expressed in plaque material because of their release into the systemic circulation. To test this assumption, we performed a comparative analysis of miRNAs purified from circulating EVs and plaque material, both obtained from identical patients undergoing surgical carotid endarterectomy. miRNA profiles were generated by high-throughput sequencing (small-RNAseq) and possible transcriptomic targets (mRNAs) of the miRNAs identified *in silico* by bioinformatic methods. By using this approach, we wanted to show that EV-associated miRNAs circulating in the vascular system are present in the plaque material and may serve as indicators for the presence of atherosclerotic plaques.

## Methods

### Patient and control group identification and selection

**Figure 1:**
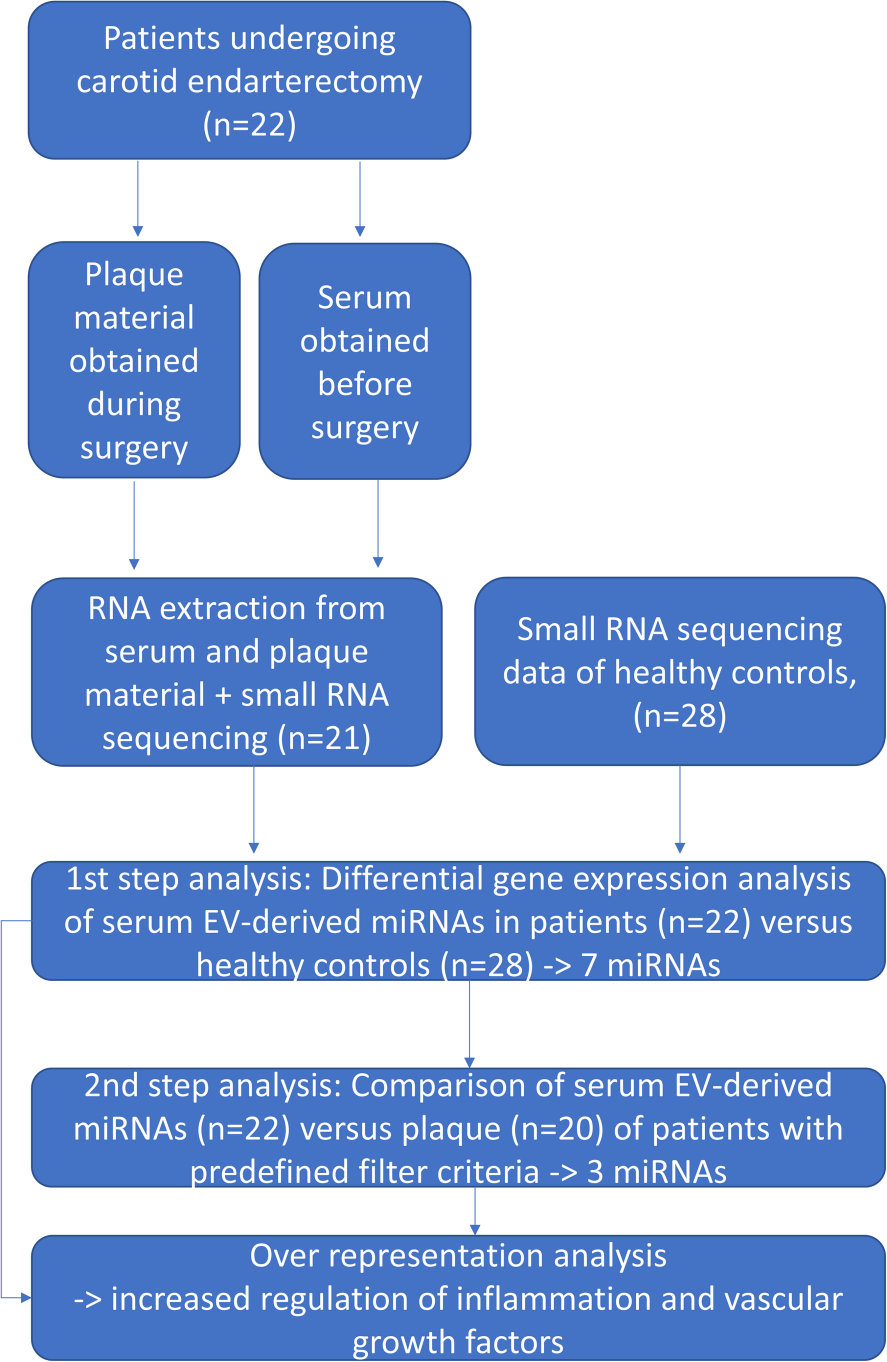
An overview of the study procedure.

Twenty-two patients who presented with atherosclerotic carotid arterial stenosis and were scheduled for carotid endarterectomy were recruited from the Departments of Vascular Surgery of the Ludwig-Maximilians-University Hospital Munich and from the InnKlinikum Altötting, Germany. Small-RNAseq data from healthy controls (*n* = 28) derived from a previously performed study (6) were used for comparing the serum and plaque miRNA profiles of patients with healthy controls. Figure 1 gives an overview of the study procedure.

Decision-making was done by following an interdisciplinary team approach (neurovascular conference) and according to available guidelines (13). In summary, patients with symptomatic stenosis of the internal carotid artery were scheduled for carotid endarterectomy whenever possible between 3 and 7 days after the onset of symptoms. Indication for surgery in clinically asymptomatic patients was seen in case of stenosis progression, high-grade bilateral stenosis (or high-grade stenosis and contralateral occlusion), silent cerebral infarction from the carotid stenosis on cerebral imaging, or clinical evidence of vulnerable plaque morphology. Age and gender were taken into consideration in the decision-making process in all patients irrespective of symptoms.

Carotid endarterectomy was performed according to standard techniques (eversion endarterectomy or carotid endarterectomy with patch closure) corresponding to anatomical and morphological criteria.

The exclusion criteria for the study were missing informed consent, age <18 years, active or chronic infections (e.g., HIV, Hepatitis B/C infection), presence of an inflammatory disorder other than atherosclerosis, malignant tumors, limited life expectancy of less than 6 months independent of atherosclerotic disease, and any kind of immunodeficiency or pharmacologic immunosuppression. Clinical and demographic data of the study populations are presented in Table 1.

**Table 1:** An overview and comparison of the demographic profiles of patients and healthy controls.

	Carotid artery stenosis (*n* = 22)	Healthy controls (*n* = 28)	*p*-Value
Demographics^a^			
Age (years)	74 (67–80)	42 (35–52)	<0.001
Height (cm)	169.5 (160.8–174.8)	180.0 (171.5–183.0)	<0.001
Weight (kg)	77.5 (72.2–88.8)	80.0 (74.5–88.5)	0.546
BMI (kg/m²)	27.0 (26.6–29.4)	25.5 (23.9–26.6)	0.004
Length of stay (days)	6.5 (6.0–9.2)	–	–
Gender (female/male)	7/15	10/18	1
Intensive care postop (no/yes)	15/7	–	–
Pre-existing risk factors^b^			
Smoking (no/yes)	13/9	–	–
Arteriosclerosis in family (no/yes)	18/4	–	–
Hypertension (no/yes)	2/20	–	–
Diabetes (no/yes)	13/9	–	–
Kidney insufficiency (no/yes)	15/7	–	–
Medications^c^			
NOAC^d^ (no/yes)	20/2	–	–
Phenprocoumon (no/yes)	21/1	–	–
Dual antiplatelet (no/yes)	19/3	–	–
Single antiplatelet (no/yes)	4/18	–	–
Oral antidiabetes (no/yes)	13/9	–	–
Insulin (no/yes)	21/1	–	–
Laboratory findings^e^			
Fasting glucose (mg/dL)	100.0 (94.0–116.0)	–	–
Cholesterol (mg/dL)	148.0 (142.0–189.0)	–	–
Cholesterol HDL (mg/dL)	43.0 (37.0–58.0)	–	–
Cholesterol LDL (mg/dL)	86.0 (70.5–112.0)	–	–
Triglycerides (mg/dL)	105.0 (85.5–163.0)	–	–
Hba1c (%)	6.1 (6.0–6.5)	–	–
Creatinine (mg/dL)	1.0 (0.7–1.2)	–	–
GFR (mL/min)	69.9 (62.0–82.2)	–	–

^a^ Values are median, Q25 and Q75.

^b^ No medical preconditions in the healthy control cohort.

^c^ Medication at admission time.

^d^ New oral anticoagulants.

^e^ Laboratory values at admission time. Values are median, Q25, and Q75.

### Sample collection

After a preoperative identification of patients according to the inclusion and exclusion criteria, consenting patients were included, and preoperative data were obtained. On the day of the surgery, blood was drawn after the induction of anesthesia through a G20 arterial line. Blood samples were centrifuged after 30 min at 2,000 × *g* for 10 min, aliquoted, and serum stored at −80°C until further processing. Plaque material was obtained during open carotid arterial endarterectomy from the identical patients. This material was placed into plastic Falcon tubes and frozen at −80°C.

### Extracellular vesicle purification, RNA extraction, depletion, library preparation, and sequencing

A quantity of 1 mL serum was processed using the miRCURY Exosome Isolation Kit (Qiagen, Venlo, Netherlands). EV purification and precipitation using this kit was performed as previously described (14). Briefly, a 400 µL precipitation buffer was added, mixed by vortexing, and incubated at 4°C for 60 min. The mixture was then centrifuged at 1,500 × *g* for 30 min to receive the pellet. After discarding the supernatant, the pellet was resuspended in a 270 µL resuspension buffer. For RNA extraction, the NucleoSpin miRNA Plasma Kit (Macherey-Nagel GmbH & Co. KG, Düren, Germany) was applied according to the supplier's manual. To achieve higher concentrations, serum RNA samples were evaporated and eluted in a smaller volume of 6 µL. Plaque material for RNA extraction was collected from different plaque sites to account for the heterogeneous material. Plaque tissue RNA extraction was performed according to the protocol described previously (15). Six microliters of serum RNA and 300 ng of plaque RNA were used as starting material for library preparation. The integrity of plaque RNA was assessed by capillary electrophoresis using the RNA 6000 Nano assay on Bioanalyzer 2100 (Agilent Technologies, Inc., Santa Clara, California, USA). Small RNA library preparation was performed using the NEBNext Multiplex Small RNA Library Prep Set for Illumina (New England Biolabs Inc., Ipswich, USA) as described previously (16). Fifty cycles of single-end sequencing on the HiSeq2500 (Illumina Inc.) were performed using the HiSeq Rapid SBS and SR Cluster kits (Illumina Inc., Eindhoven, Netherlands). The serum samples of healthy controls underwent exactly the same type of processing.

### Bioinformatic analysis

The processing of raw data and alignment of reads were performed as previously described (14). The sequencing data of all 22 serum EV samples but only of 20 plaque samples were included for subsequent differential gene expression (DGE) analyses performed using DESeq2 (Version 1.30.1) (17). Technical variations resulting from multiple sequencing runs were accounted for in the model. The Benjamini–Hochberg method controlling the false discovery rate was applied to reduce type I error accumulation. The regulatory impact of miRNAs was assessed by identifying experimentally validated mRNA targets with miRTarBase (18) and performing an overrepresentation analysis of reactome pathways (19) using clusterProfiler (Version 4.10.0) (20).

A DGE analysis was performed for the serum EV–derived miRNA profiles of patients and healthy controls by applying the following filter criteria: the absolute value of log_2_fold change (log_2_FC) < −1 (=higher miRNA expression in patient serum compared with control serum), an adjusted *p*-value (*p*_adj_) < 0.05, group mean expression (ME) > 10, and ME_patient-serum _> ME_control-serum_.

The resulting miRNAs were further examined in the DGE analysis by comparing the serum EV-derived and plaque-derived miRNA profiles in patients. Only miRNAs that fulfilled the criteria of log_2_FC < 0 (=higher miRNA expression in plaque compared with serum), ME > 10, and ME_plaque _> ME_patient-serum_ were included in this analysis.

### Statistical analysis of demographics and clinical data

As no data on group sizes for making a comparison between miRNA expression values in EVs vs. plaque material were available, the sample size calculation was done based on a previous study by our group that included 24 patients with carotid artery stenosis, and the EV miRNA expression values were compared with those in 28 healthy controls. An overlap analysis (DGE) resulted in the identification of seven differentially regulated miRNAs between both groups (6). We assumed that a comparable group size may also be required for the detection of miRNAs regulated in plaque material vs. circulating miRNAs and therefore included 22 patients undergoing carotid artery surgery.

The demographic and clinical data of the groups were compared using the non-parametric Mann–Whitney *U*-test. The Chi-square test was applied for comparing categorical variables. Data analysis was performed using Python version 3.9 (Python Software Foundation, Beaverton, USA). The Python packages and versions used were Pandas (1.3.3), NumPy (1.21.2), Matplotlib (3.4.3), and SciPy (1.6.3). Data in the text and in tables are reported as median and interquartile range (IQR). All statistical tests were two-tailed, and a *p*-value < 0.05 was considered statistically significant.

### Availability of data and materials

The data generated in this work will be stored in the European Nucleotide Archive (ENA, PRJEB72543). Unfiltered DGE lists of the two types of comparisons made are shown in the Supplementary Tables E1, E2. Principal component analysis (PCA) figures are shown in the Supplementary Figures E1, E2.

### Ethics approval, patient recruitment, and participatory consent

The study was approved by the Ethics Committee of the Medical Faculty of the Ludwig-Maximilians-University of Munich, Germany, and registered under protocol #17-572. This work was performed in accordance with the Declaration of Helsinki. Written informed consent was obtained from the participants and all study samples were pseudonymized.

## Results

### Comparison of patient demographics with healthy controls and bioinformatic analysis

Healthy controls were significantly younger and had a slightly lower body mass index (BMI) than the patient group. The factors of gender distribution and weight were comparable between the two groups (see Table 1).

The sequencing results were of high quality for all serum EV samples (raw sequencing data available in ENA). Of the 22 plaque samples, the sequencing results of two were excluded because of a largely deviated sequencing profile with a high amount of degraded reads and minimal library size. In the PCA of the top 500 genes sorted by variance, the plaque material showed a clear separation from the serum-derived EV samples on the first component, explaining the over 33% of the total variance in the data set. The serum-derived EV samples revealed that two clusters on the third principal component could be defined separating the patients from the healthy controls; however, both clusters showed a significant overlap and some samples deviated from their clusters (see Supplementary Figures E1, E2).

### Serum miRNA profile of patients undergoing carotid arterectomy vs. healthy controls and the associated signaling network

**Figure 2:**
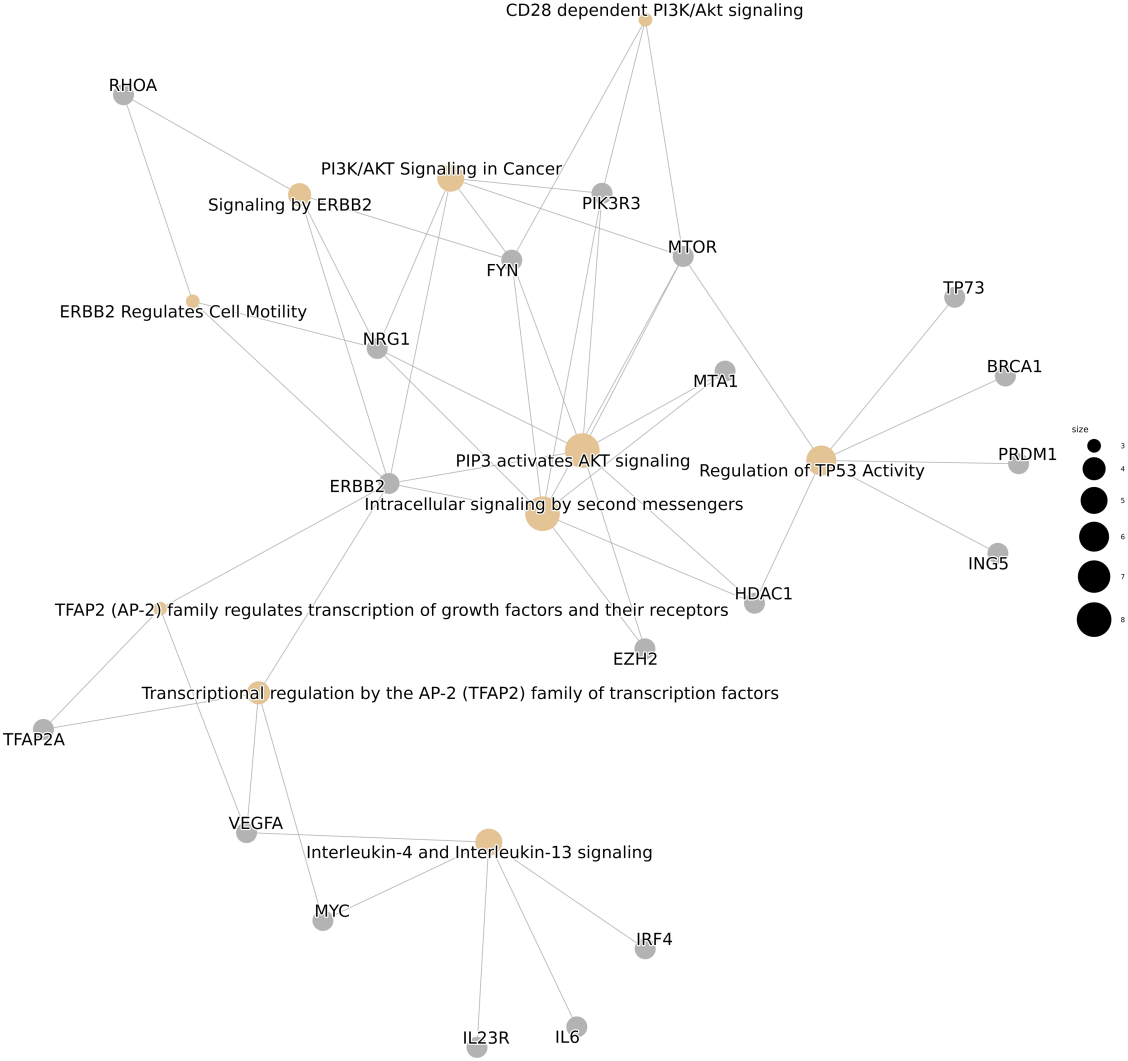
An overrepresentation analysis of reactome pathways.

Small-RNA sequencing was successful in all analyzed patients. The DGE analysis of patient serum compared with that of healthy controls, and patient serum compared with patient plaque material, led to the detection of 729 miRNAs of 2,292 human miRNAs with a BaseMean (normalized mean miRNA expression over all samples) > 1. As a first step, we compared serum miRNA profiles between carotid artery patients and healthy controls by using DGE (step 1) with applied filter criteria of an absolute value of log_2_fold change (log_2_FC) < −1 (=higher miRNA expression in patient serum compared with control serum), an adjusted *p*-value (*p*_adj_) < 0.05, mean expression (normalized mean miRNA expression over all samples of the respective group; ME) > 10, and ME_patient-serum _> ME_control-serum_. This comparison revealed seven significantly upregulated miRNAs in patient EVs derived from serum (see Table 2). The complete and unfiltered DGE lists are available in the Supplementary Tables E1, E2. An overrepresentation analysis of experimentally validated mRNA targets of these seven miRNAs revealed an increased regulation of inflammation and vascular growth factors, key players in artherosclerosis and plaque formation (21, 22). The most significantly enriched pathways included Interleukin-4 and Interleukin-13 signaling (R-HSA-6785807), platelet activation, signaling and aggregation (R-HSA-76002), signaling by interleukins (R-HSA-449147), the VEGFA-VEGFR2 pathway (R-HSA-4420097), and signaling by VEGF (R-HSA-194138), among others (Figure 2).

**Table 2:** Significantly differentially expressed miRNAs in patient serum–derived EVs compared with serum-derived EVs of healthy controls and their presence in plaque material.

miRNA	Comparison patient serum vs. healthy control serum^a^				Comparison patient serum vs. patient plaque material^b^			
	log_2_FC	Adjusted *p*-value	ME_control serum_	ME_patient serum_	log_2_FC	Adjusted *p*-value	ME_patient serum_	ME_patient plaque_
**miR-193b-5p**	**−1**.**761**	**0**.**009**	**36**.**19**	**50**.**18**	**−0**.**908**	**0**.**070**	**50**.**18**	**50**.**23**
miR-3168	−1.620	0.028	1,570.93	2,379.33	−1.181	0.195	2,379.33	0.43
miR-320c	−1.319	0.014	221.92	241.44	−0.210	0.713	241.44	88.57
miR-320d	−1.297	0.019	109.93	145.14	−0.577	0.203	145.14	106.76
**miR-193a-5p**	**−1**.**246**	**0**.**004**	**298**.**19**	**468**.**18**	**−1**.**361**	**1.18 × 10^−5^**	**468**.**18**	**905**.**07**
miR-320b	−1.104	0.019	694.23	904.82	−0.122	0.856	904.82	494.89
**miR-125a-3p**	**−1**.**042**	**0**.**019**	**10**.**81**	**26**.**61**	**−0**.**499**	**0**.**083**	**26**.**61**	**41**.**67**

miRNAs fulfilling filter criteria in both comparisons are printed in bold.

^a^ log_2_fold change (log_2_FC) < −1 (=higher miRNA expression in patient serum–derived EVs compared with control serum–derived EVs), an adjusted *p*-value (*p*_adj_) < 0.05, group mean expression (ME) > 10, and ME_patient-serum _> ME_control-serum._

^b^ log_2_FC < 0 (=higher miRNA expression in plaque compared with serum-derived EVs as baseline), ME > 10, and ME_plaque _> ME_patient-serum._

### Serum miRNA profile vs. plaque miRNA profile of patients undergoing carotid endarterectomy

In a second step (step 2), a comparison by DGE analysis was performed between the serum miRNA of the first DGE analysis (step 1) and the plaque miRNA profile of patients using the filter criteria of log_2_FC < 0 (=higher miRNA expression in plaque compared with serum as baseline), ME > 10, and ME_plaque _> ME_patient-serum_. The analysis showed that three (miR-193a-5p, miR-193b-5p, and miR-125a-3p) of seven miRNAs fulfilling these filter criteria were significantly upregulated (*p* < 0.05) in both compartments (see Table 2).

## Discussion

As atherosclerosis is an extremely common disorder and major complications often occur because of late diagnosis, biomarkers for early plaque detection are of urgent need.

In this study, we tested the hypothesis that miRNAs present in circulating EVs are also expressed in plaque material and thus may serve as biomarkers for detecting the presence of plaques. EV-associated miRNAs are well protected by the EV envelope and thus may present more stably expressed circulating biomarkers. This approach would therefore allow the adoption of a robust liquid biopsy technique to identify patients with atherosclerotic plaques and would be advantageous over direct plasma/serum sampling.

When comparing miRNA expression values between EVs and plaques from identical patients undergoing carotid endarterectomy, we identified *miR-193a-5p*, *miR-193b-5p*, and *miR-125a-3p* in both compartments (Table 2). An overexpression of *miR-193a-5p* in EVs was also seen in a different cohort of patients with symptomatic carotid artery stenosis in a previous study by our group (6).

Moreover, a study by Jha et al. identified apolipoprotein L1 (APOL1) as the miR-193a-5p target. APOL1 as the major apoprotein of high-density lipoproteins (HDL) is, among other tissues, present in endothelial cells and in blood circulation (23, 24). It is generally known that HDL protects against atherosclerosis (25). An upregulated miR-193a-5p in EVs and plaques implies a reduced APOL1, which ultimately promotes atherosclerosis. Another miRNA of the miR-193 family, miR-193b-5p, which was also upregulated in the plaques of our cohort was shown to have increased expression when endothelial cells were exposed to oxidized low-density lipoproteins (a hallmark of oxidative stress). Dègano et al. not only identified miR-193b-5p as part of this atherosclerosis-relevant mechanism but could also validate miR-193b-5p in patients' serum by qPCR (26). The third interesting miRNA found in our study, *miR-125a-3p,* was shown to regulate the function of vascular smooth muscle cells when expressed in EVs derived from endothelial cells, which finally led to a progression of atherosclerosis. When systemic inflammation was simulated in this cellular model by administering lipopolysaccharide (LPS), the expression of *miR-125a-3p* increased and mediated the proliferation and migration of vascular smooth muscle cells. In addition, *miR-125a-3p* was also shown to regulate VEGFA (27), a major player in atherosclerotic angiogenesis and plaque progression (28)*.* Interestingly, the administration of curcumin and nicotinic curcumin upregulated the expression of this miRNA, suggesting therapeutic usefulness in atherosclerosis, probably mediated by an anti-inflammatory effect (29).

There are numerous published studies available that have investigated circulating miRNAs from serum and plasma in patients suffering from atherosclerosis, and some examples are (30, 31). A few reports on miRNA analysis in plaque material are also available (32, 33). However, these studies investigated only a few preselected miRNAs and did not have paired samples of blood specimens to compare with. Unlike our study, the studies of both Cipollone Maitrias performed a patient subgroup comparison (symptomatic vs. asymptomatic). However, none of the miRNAs mentioned in the reviews and the reports about plaque miRNAs were found in our cohort. This discrepancy is probably due to the investigation of different blood component sources and partly due to (in the meanwhile) an outdated methodology. Unlike the mentioned reports focusing on the miRNAs of plasma, serum, or cellular origin, more recent studies investigated EV-associated miRNAs derived from endothelial, vascular smooth muscle, and dendritic cells, as well as from platelets and monocytes/macrophages in the context of atherosclerosis (34). A pivotal advantage of miRNAs associated with EVs is that their presence is prolonged in blood circulation (35–37). In our study, our comparison of circulating EV miRNAs with plaque-derived miRNAs of the same patient allowed us to follow a very stringent filtering step, which certainly can be considered a strength of this study. To our knowledge, such a paired analysis has not been reported in the literature.

We would also like to mention that the next generation sequencing data in this study revealed several highly upregulated miRNAs in plaque material compared with serum-derived EVmiRNAs of healthy controls and patients (see Supplementary Table E2). These miRNAs may play a role in plaque formation and maintenance, but their analyses are beyond the scope of this work.

A limitation of our study results from the fact that the identified miRNAs were isolated from circulating serum EV precipitates. The resulting EV pellets contained cell-free proteins as coisolates, which may include carriers of circulating serum miRNAs (e.g., argonaut 2 or lipoprotein particles) (38, 39). Some of the miRNAs identified in our study may therefore coprecipitate from serum and were not exclusively derived from EVs. Another limitation is the significant discrepancy in age between the patient cohort and the healthy controls. We were not able to obtain samples from healthy older age individuals.

## Conclusion

Our study on patients with symptomatic carotid artery stenosis undergoing surgery identified several miRNAs present in both circulating EVs and plaque material. Among these, the miRNAs *miR-193b-5p*, *miR-193a-5p*, and *miR-125a-3p* fulfilled stringent selection criteria and showed a comparable direction of regulation in both compartments. These findings suggest the usefulness of EV-associated miRNAs as biomarkers for detecting plaque presence and may indicate the need for early surgical intervention in clinically asymptomatic patients ahead of the occurrence of adverse events.

## Data Availability

The datasets presented in this study can be found in online repositories. The names of the repository/repositories and accession number(s) can be found below: The data generated in this work will be stored in the European Nucleotide Archive (ENA, PRJEB72543), https://www.ebi.ac.uk/ena/browser/view/PRJEB72543. Unfiltered DGE lists of the two comparisons are shown in the Supplementary Tables E1, E2. PCA figures are shown in the Supplementary Figures E1, E2.

## Supplementary material

Supplementary Table E1

An unfiltered list of DGE analysis comparing the EV-miRNA profiles of patient serum with those of healthy controls. baseMean = normalized mean expression over all samples; *p*_adj_ = adjusted *p*-value.

Supplementary Table E2

An unfiltered list of DGE analysis comparing the EV-miRNA profiles of patient serum with the miRNA profiles of plaque material. baseMean = normalized mean expression over all samples; *p*_adj_ = adjusted *p*-value.

Supplementary Figures E1 and E2

A PCA of the 500 gene showing the highest variance in the dataset. The relative explained variance of each principal component is given on each axis. The plaque samples show a clear separation from serum-derived EV samples on the first principal component, while the third principal component is seen separating healthy serum EVs from patient serum EVs.
