# Supplementary figures and images for: Identification of microRNA biomarkers simultaneously expressed in circulating extracellular vesicles and atherosclerotic plaques
Source: Front Cardiovasc Med. 2024 Apr 25;11:1307832. doi: 10.3389/fcvm.2024.1307832 (PMC11079260; doi:10.3389/fcvm.2024.1307832)

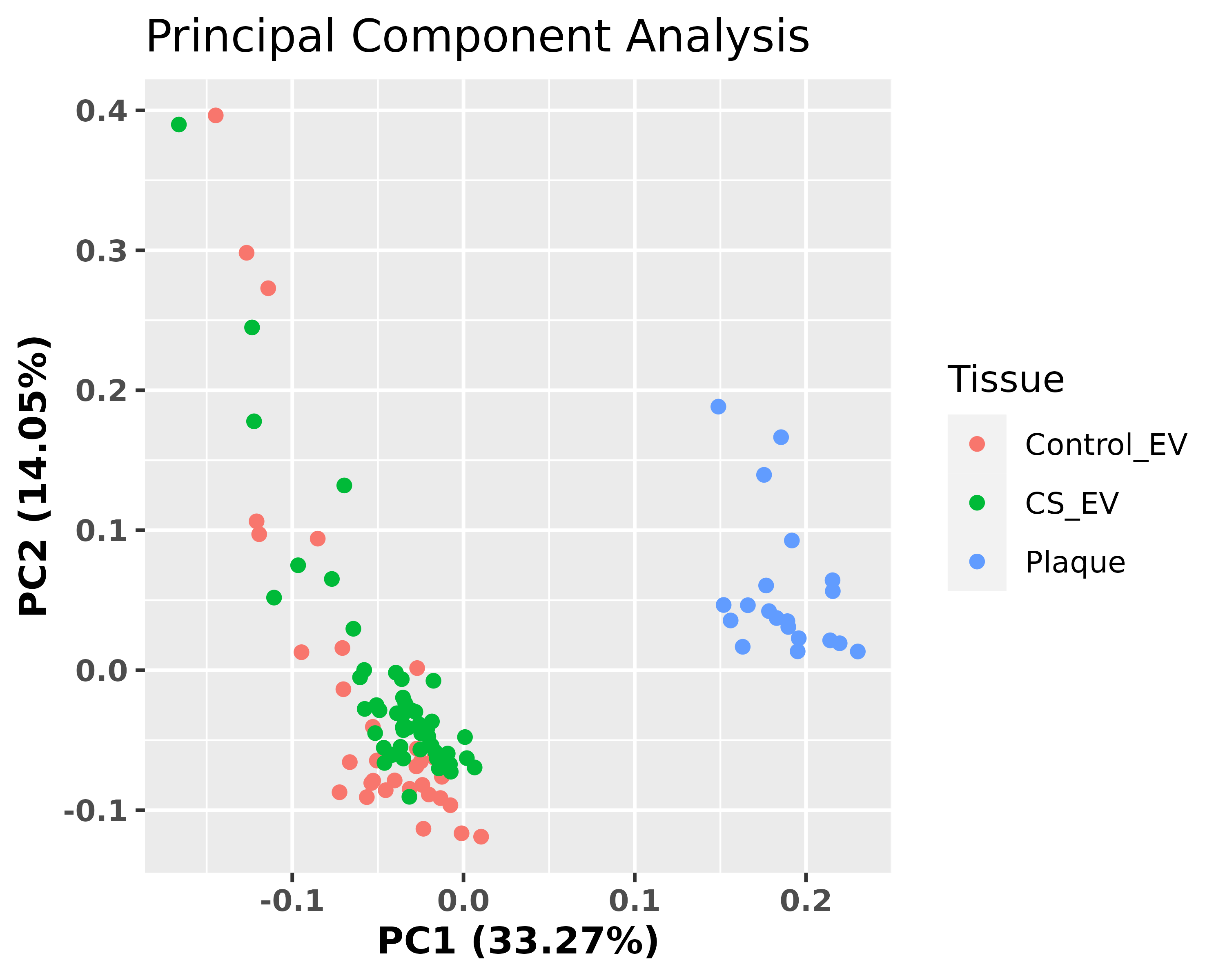

Supplement: Supplementary file 1 [file Image1.png]

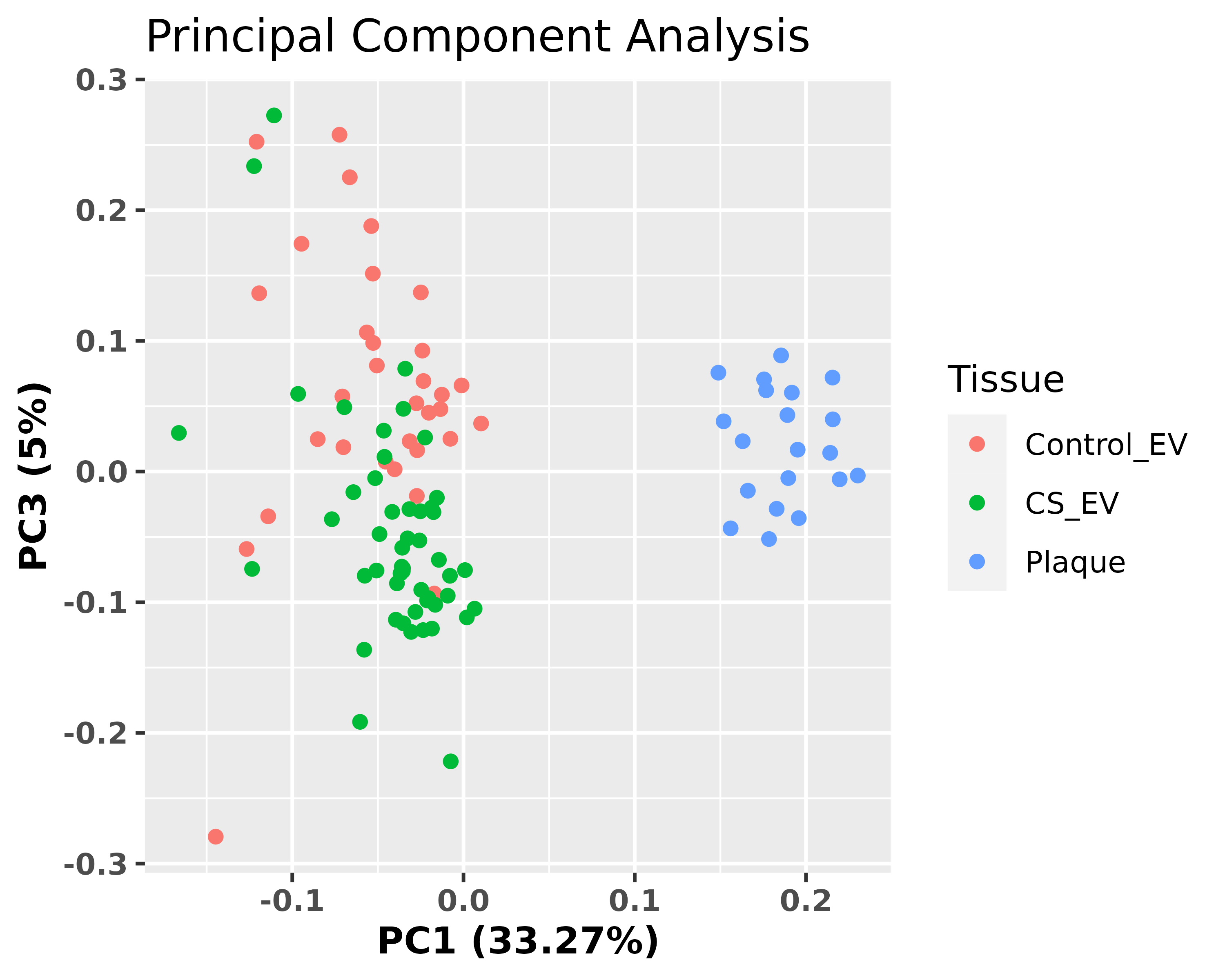

Supplement: Supplementary file 2 [file Image2.png]
